# Experimental Models for Studying Structural and Functional State of the Pathological Liver (Review)

**DOI:** 10.17691/stm2023.15.4.06

**Published:** 2023-07-28

**Authors:** D.P. Krylov, S.A. Rodimova, M.M. Karabut, D.S. Kuznetsova

**Affiliations:** Laboratory Assistant, Scientific Laboratory of Molecular Biotechnologies, Research Institute of Experimental Oncology and Biomedical Technologies; Privolzhsky Research Medical University, 10/1 Minin and Pozharsky Square, Nizhny Novgorod, 603005, Russia; Student, Institute of Biology and Biomedicine; National Research Lobachevsky State University of Nizhny Novgorod, 23 Prospekt Gagarina, Nizhny Novgorod, 603022, Russia; Junior Researcher, Laboratory of Regenerative Medicine, Scientific Laboratory of Molecular Biotechnologies, Research Institute of Experimental Oncology and Biomedical Technologies; Privolzhsky Research Medical University, 10/1 Minin and Pozharsky Square, Nizhny Novgorod, 603005, Russia;; Researcher, Laboratory of Genomics of Adaptive Antitumor Immunity, Research Institute of Experimental Oncology and Biomedical Technologies; Privolzhsky Research Medical University, 10/1 Minin and Pozharsky Square, Nizhny Novgorod, 603005, Russia;; Head of Laboratory of Molecular Biotechnologies, Research Institute of Experimental Oncology and Biomedical Technologies; Privolzhsky Research Medical University, 10/1 Minin and Pozharsky Square, Nizhny Novgorod, 603005, Russia; Head of the Research Laboratory for Molecular Genetic Researches, Institute of Clinical Medicine; National Research Lobachevsky State University of Nizhny Novgorod, 23 Prospekt Gagarina, Nizhny Novgorod, 603022, Russia

**Keywords:** experimental models, liver pathology, *in vivo*, *in vitro*, *ex vivo*

## Abstract

Liver pathologies remain one of the leading causes of mortality worldwide. Despite a high prevalence of liver diseases, the possibilities of diagnosing, prognosing, and treating non-alcoholic and alcoholic liver diseases still have a number of limitations and require the development of new methods and approaches. In laboratory studies, various models are used to reconstitute the pathological conditions of the liver, including cell cultures, spheroids, organoids, microfluidic systems, tissue slices.

We reviewed the most commonly used *in vivo*, *in vitro*, and *ex vivo* models for studying non-alcoholic fatty liver disease and alcoholic liver disease, toxic liver injury, and fibrosis, described their advantages, limitations, and prospects for use. Great emphasis was placed on the mechanisms of development of pathological conditions in each model, as well as the assessment of the possibility of reconstructing various key aspects of pathogenesis for all these pathologies.

There is currently no consensus on the choice of the most adequate model for studying liver pathology. The choice of a certain effective research model is determined by the specific purpose and objectives of the experiment.

## Introduction

Liver pathologies continue to be one of the leading causes of mortality worldwide. Such diseases as non-alcoholic fatty liver disease (NAFLD) and its progressive form, non-alcoholic steatohepatitis, acute toxic damage, and alcoholic liver disease (ALD) are socially important [1]. NAFLD is characterized by abnormal accumulation of triglycerides (TG) in hepatocytes and is considered to be the most common type of chronic liver disease [2]. Conditions associated with alcohol abuse are also common. According to the “Global status report on alcohol and health” (2018) published by the World Health Organization, 3 million people die annually from alcohol abuse worldwide [3]. Besides, both NAFLD and ALD are often accompanied by extrahepatic complications including cardiovascular diseases and malignant neoplasms [4]. Despite a wide prevalence of liver diseases, the possibilities of diagnosis, prognosis, and therapy of NAFLD and ALD are still limited and require new methods and approaches [5].

In laboratory studies, various models are used to reconstitute pathological liver conditions, with their choice depending on specific goals and tasks of the experiment, for example, *in vitro*, *ex vivo*, and *in vivo* models. Each model has its merits and drawbacks, which are to be taken into consideration when conducting a scientific experiment [6].

**Figure 1.**
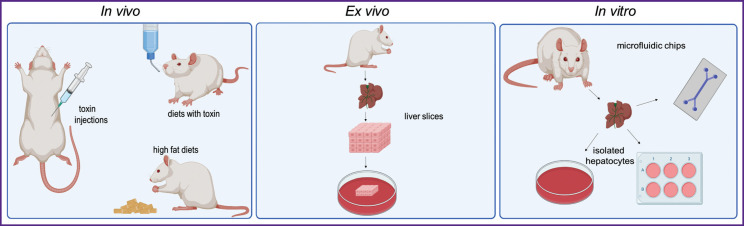
Models of liver pathologies

*In vivo* models imply the work with experimental animals. The spectrum of animals used for modeling liver pathology varies from fish (*Danio rerio*) to primates. However, the representatives of the *Rodentia* order are used as model animals in the majority of biomedical investigations (Figure 1) [7]. The *in vivo* models serve as a gold standard of laboratory investigations and are preferable for the reconstruction of complex pathogenic and pathophysiological processes [8]. However, they are not suitable for the studies where it is important to exclude the individual body response or for the series of experiments with large databases since these researches require a great number of animals and are related with high costs and labor effort [9].

The development of cellular and molecular biology made it possible to implement *in vitro* models into practice, for example, cell lines, spheroids, organoids, “organs-on-a-chip” [10]. Primary or isolated hepatocytes are used as *in vitro* models. The experiments on the *in vitro* models made investigations less expensive, reproducibility faster; they became less time-consuming as compared to the *in vivo* investigations. Moreover, the application of cell cultures allowed to overcome the ethical problem, which is certainly a great advantage of these models over the animal ones [11]. However, primary and isolated hepatocytes are phenotypically unstable and do not have intratissue interactions which occur in the native organ [12]. The microfluidic technology, which allows for the usage of more complicated models and simulation of the native conditions of life activity for the cells and tissues, is a promising option of using *in vitro* models. This technology gives the possibility to test the effect of various toxic and medicinal agents on the hepatic cells [13].

An intermediate model between *in vitro* and *in vivo* is the *ex vivo* living tissue (slices) transferred from the organism to the external artificial environment. Such a model represents sections of tissue or tissue slices, which preserve the native architecture and heterogeneity of the tissue. At the same time, the main intercellular interactions are maintained and active metabolic pathways are preserved in these slices, which allows specialists to study targetedly the processes running at the organ level [14]. However, the period of liver slices viability is usually about three days, which prevents long-term investigations [15]. Besides, there is no unified protocol for harvesting the liver slices and their subsequent culturing at present [16], making the results irreproducible.

Thus, presently there is no agreement about the choice of the most appropriate model for studying liver pathology since each has its benefits and drawbacks. In this review, we consider the most frequently used models and describe their merits, limitations, and further prospects for application.

## *In vivo* models

### Alcohol liver disease

An animal ALD model makes it possible to study in detail the mechanisms of the disease initiation and progression. These models are used to explore potential therapeutic targets for ALD treatment [17].

Bertola et al. [18] simulated toxic liver damage in mice using liquid ethanol-containing Lieber–DeCarli diet for 10 days with subsequent administration of a single ethanol dose, which led to marked neutrophilia and granulocyte infiltration as well as to extensive damage and fatty dystrophy of the hepatic tissue. When analyzing the biochemical blood indicators, a significant rise of the alanine aminotransferase and aspartate aminotransferase level was observed. Owing to a simple and reproducible protocol, this model is presently widely used for studying early stages of ALD and alcoholic hepatitis. Later, Gao et al. [19] modified the initial model to study a chronic course of ALD. They modeled alcoholic damage for 12 weeks to develop steatohepatitis and moderate fibrosis. The authors [20] described the protocol combining high consumption of ethanol and transfer of the animals to a high-fat diet. This protocol induced the development of severe steatohepatitis with neutrophil infiltration and weakly expressed fibrosis. Khanova et al. [21] have designed a protocol of intragastric infusion of high ethanol doses to mice in combination with the diet with a high content of fatty acids and cholesterol. In this model, expressed infiltration of the liver tissue with neutrophils and fibrosis formation also take place.

**Figure 2.**
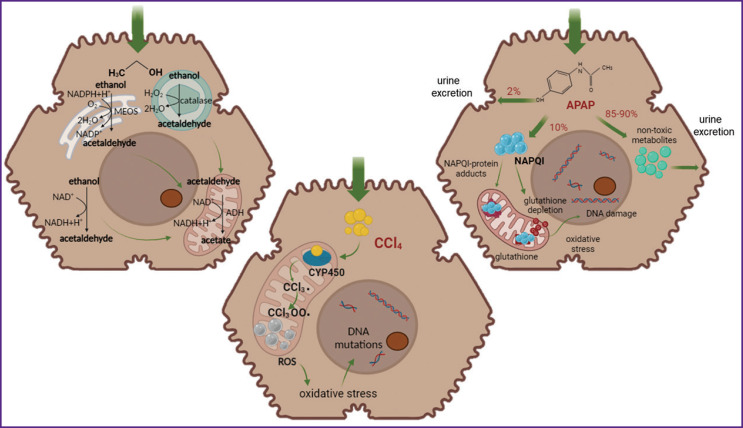
Mechanisms of toxic action of various agents

The *in vivo* models helped identify numerous mechanisms underlying ALD pathogenesis, alcohol-associated steatohepatitis, establish the pathogenetic role of microRNA, autophagia, and extracellular vesicles [19]. The key aspects of the pathological process caused by the direct effect of ethanol and its metabolites on the liver and other organs have been found. Ethanol is metabolized in the liver by alcohol dehydrogenase enzyme (ADH), which leads to the production of the toxic metabolite acetaldehyde and the reduced NAD (nicotinamide adenine dinucleotide) equivalents. Acetaldehyde causes oxidative stress in the liver and the disturbance of mitochondrial function [22], while an increased production of NAD impairs the redox balance and the processes of gluconeogenesis [23]. Ethanol oxidation also involves cytochrome P450-depending microsomal ethanol-oxidizing system and catalase (Figure 2). Chronic exposure to ethanol decreases the metabolic activity of hepatocytes, which is accompanied by the disturbance of liver detoxification function. At the cellular level, dysfunction of mitochondria and endoplasmic reticulum, reduction of proteasome activity, impairment of vesicular hepatocyte transport could be observed [24]. The cumulative effect of the ethanol-induced stress of the organelles in hepatocytes increases the sensitivity of the cells to death by the apoptotic or necrotic pathway [25].

Petrasek et al. [26] have shown that activation of innate immunity is one of the key aspects of the ALD pathogenesis, particularly in alcohol hepatitis. The production of proinflammatory cytokines promotes fast progression of the disease. The direct impact of ethanol and its metabolites on hepatocytes results in endoplasmic reticulum stress, induces IRF3, which, apart from activation of type I interferon, triggers the mitochondrial pathway of apoptosis. It leads to the release of the so-called molecular fragments, DAMPs (damage-associated molecular patterns), particularly of uric acid, adenosine triphosphate acid (ATP), HMGB1.

Tsukamoto et al. [27] designed a rat ALD model including direct introduction of ethanol and nutrients via the cannula implanted into the animal stomach. This model gives the possibility to achieve a high ethanol concentration in blood due to a strict control of the ethanol dose consumed by the animals. In ALD induced by this model, one could observe expressed steatosis, formation of megamitochondria, central lobular and pericellular fibrosis, central necrosis, and mixed inflammatory infiltrate. These pathological changes are also typical for the ALD course in humans [28].

All *in vivo* models have some drawbacks, since they do not reflect completely the specificity and complications observed in alcohol damage in people. Owing to the fast ethanol metabolism in rodents, severe ALD forms do not usually develop even in its long-term effect [29]. This fact is explained by a low susceptibility of the model animals to ethanol, which does not allow to reliably reproduce human ALD pathogenesis. To solve the problem, some authors use the combination of ethanol and other toxins (lipopolysaccharide, tetrachloromethane (CCl4), concanavalin A) in order to induce a severe form of liver damage [30]. However, even the combined models only partly reproduce the ethanol action, since the majority of the changes observed are caused by the secondary effects of other toxic agents rather than the primary action of ethanol hepatotoxicity [31].

### Liver fibrosis

#### Induction by tetrachloromethane

Tetrachloromethane (CCl4) is used most commonly to model fibrosis and cirrhosis of the liver in rodents. The CCl4 toxicity has a complex character, its effects are different depending on the dose and action duration. Low doses usually cause transitory effects associated with the impairment of Ca^2+^ transport, lipid homeostasis, cytokines release, and triggering apoptosis with subsequent regeneration. High doses or long-term exposure result in extensive liver damage including fatty dystrophy, fibrosis, cirrhosis, and hepatocellular carcinoma. Besides, at high doses and the level of hepatocellular necrosis exceeding the regenerative liver capacity, hepatic failure develops with a high risk of fatal outcome. Marginal doses of CCl4 lead to nonspecific toxicity with depression of the central nervous and respiratory systems [32].

Tetrachloromethane metabolism occurs with the involvement of cytochrome P450 2E1 (CYP2E1), a heme-containing enzyme participating in detoxification of xenobiotics. Heme iron in CYP2E1 makes it possible to reduce directly, for example, carbon dioxide with formation of reactive oxygen species (ROS) and conversion of xenobiotics into toxic products [33]. The enzyme activity leads to the generation of trichloromethyl and trichloromethylperoxyl radicals, which are involved in the free radical reactions and the dependent processes of lipid peroxidation [34]. Trichloromethyl radical reacts with various cellular macromolecules [35] and promotes hydrogen abstraction from unsaturated fatty acids with toxic chloroform formation. Accumulation of active radicals initiates acute phase of inflammation with derangement of the membrane-associated proteins, inhibition of the protein synthesis activity, release of the cytoplasmic enzymes into blood, and nucleotide destruction. This chain of events leads eventually to the activation of Kupffer cells and extensive centrilobular necrosis of hepatocytes [36] (see Figure 2). Besides, production of cytokines by the immune cells facilitates activation of the hepatic stellate cells and the generation of myofibroblasts, which trigger excessive collagen deposition in the intercellular space (the process of fibrotic scarring) [37].

Modeling of toxic liver damage with tetrachloromethane is carried out both on rats and mice. Susceptibility of animals to CCl4-induced liver fibrosis depends on their line. For example, C57BL/6 and DBA/2J mice develop fibrosis faster than BALB/c mice [38]. According to the protocol by Constandinou et al. [39], which is a gold standard for laboratory investigations, CCl4 is injected intraperitonially 2–3 times a week for 4–6 weeks at the concentration range of 300–1000 μl/kg. As an alternative, CCl4 can be introduced orally, subcutaneously, or by inhalation twice a week for 10 weeks [40]. Notably that a question about oral CCl4 introduction remains debatable. Thus, some authors state that this approach demonstrates a high speed of fibrosis induction at acceptable indicators of animal survival [41], whereas others do not recommend oral introduction unless there is absolute necessity due to high indicators of early mortality [42].

#### Induction by paracetamol

Paracetamol (N-acetyl-para-aminophenol, acetaminophen) is a nonsteroidal anti-inflammatory drug [43] used as an analgesic and antipyretic medicine [44]. In therapeutic doses, paracetamol is safe, causes no damage to the gastrointestinal tract, and has no adverse cardiorenal effects. However, if taken in a single dose exceeding 150 mg/kg body weight, hepatotoxic and nephrotoxic liver damage may be diagnosed [45]. An overdosage causes liver necrosis and failure, development of oxidative stress, which is accompanied by lipid peroxidation, oxidation of DNA and proteins, and reduction of antioxidant defense [46]. In the process of metabolism with the participation of cytochrome P450, a toxic metabolite N-acetyl-p-benzoquinone imine (NAPQI) is generated, which conjugates with the reduced glutathione even at low concentrations of the preparation [47]. In case of its high dose or glutathione depletion, NAPQI reacts with cellular proteins causing oxidative stress, lipid peroxidation, generation of free radicals, and mitochondrial damage, involving necrotic or apoptotic death of hepatocytes [48] (see Figure 2).

The *in vivo* modeling of paracetamol toxic effect received a wide application in laboratory practice using various animals: rabbits, mice, rats [49]. At present, the role of oxidative stress, ROS, and nitrogen in the paracetamol-associated hepatotoxicity is being actively studied.

Paracetamol in the dose of 200 mg/kg b.w. has been shown to significantly increase the concentration of hydrogen peroxide in the liver of C57BL/6J mice [44], whereas a single injection of the drug in the dose of 3 g/kg b.w. induces the generation of hydroperoxides in the liver [50]. Arafa et al. [51] have found that paracetamol in the dose of 1 g/kg b.w. enhances the production of nitrogen oxide in the mouse liver. In case of induced liver damage by a single introduction of paracetamol at the concentration of 1, 2, or 3 g/kg b.w., the level of nitrogen oxide in the blood and liver cells was significantly increased [52].

In almost all cases, paracetamol induces lipid peroxidation: increased concentrations of malonyldialdehyde (MDA is a secondary product of lipid peroxide) were observed in the blood serum of the rabbits receiving the drug (1 g/kg b.w.) for 9 days [53]. In rats, the effect of various doses of paracetamol was also accompanied by the increased MDA level in the blood serum and liver tissue [54]. Such elevation of intermediate peroxidation products correlated with the level of antioxidant system inhibition, i.e. with the reduced level of glutathione and enzymes participating in its metabolism, and the level of superoxide dismutase and catalase [55]. Similarly, lipid peroxide in the liver or blood serum of the animals is observed in case of the long-term introduction of lower doses of paracetamol (100 and 250 mg/kg b.w.) [56]. The effect of the paracetamol metabolites on cellular lipids is not presently studied enough and deserves more close attention.

Reactive oxygen species also affect DNA with formation of adducts, one of which is 8-hydroxydesoxyguanosine (8-OHdG) [57]. Wang et al. [58] have demonstrated that a long-term impact of 400 mg/kg b.w. paracetamol elevates considerably the 8-OhdG concentration in mice blood serum, however, when a single high dose of the preparation is received, oxidative damage to the DNA is not practically observed. One more target for the oxidative damage is proteins, which are transformed to the carbonylated form [59]. Abdul Hamid et al. [45] have found that the effect of 750 mg/kg b.w. dose of paracetamol on the rats for 7 days increases the formation of carbonyl proteins in blood plasma and kidneys causing nephrotoxicity apart from the damage to the liver tissue.

The investigations related to the activation of the immune system under the action of high paracetamol dose have shown hyperproduction of proinflammatory cytokines. The 2 g/kg b.w. dosage of paracetamol in rats increases the level of serum TNF-α, expression of *tnf-α* gene in the liver [60], the levels of IL-1β in the blood serum and liver, and the IL-6 levels in plasma [61]. Similarly, the smaller doses of paracetamol significantly elevate the level of proinflammatory cytokines in rats and mice. After the effect of paracetamol on rats (1 g/kg b.w.) or mice (0.3, 0.9, or 1.5 g/kg b.w.), the levels of TNF-α, IL-1β, and IFN-γ grow in the blood serum and liver in all animals [62]. A hyper-expression of *tnf*-*α* and *il-1β* genes in the murine liver is also observed [63].

### Steatosis

At present, experimental animals are administered diets with increased content of fats to induce NAFLD and its progressive forms [64]. Susceptibility of the rodents to modeling of fatty liver disease using food ingredients depends on their line. The C57BL/6 mice are more susceptible to NAFLD development by diet correction than BALB/c mice [65]. Primates, rhesus monkey in particular, were also offered for NAFLD study due to their genetic similarity to humans. However, these models are rarely used because of the ethical aspects and high costs of such researches. Wistar or Sprague Dawley rats are usually used for steatosis induction [66].

A classical diet for induction of nonalcoholic steatohepatitis includes a high content of sugar (40%) and fat (10%) with a deficit of essential amino acids: methionine and choline [67]. Choline is a precursor of phosphatidylcholine, which is necessary for the production of very-low-density lipoproteins [68], a complex providing transport of cholesterol and TG from the liver to the adipose tissue and muscles [69]. Methionine is necessary for glutathione synthesis, one of the main components of cellular antioxidant defense [70]. After the transition to the high-fat diet, there occurs an oxidative stress and elevation of proinflammatory cytokine and adipokine levels, which results eventually in the liver tissue damage [71]. A certain role in the steatosis pathogenesis is played by Kupffer cells, which are the first to respond to the hepatocyte damage increasing the TNF-α production and promoting monocyte recruiting [72]. Besides, Kupffer cells contribute to the enhancement of some proinflammatory pathways and mediator synthesis including NF-kB, ICAM-1, cyclooxygenase 2, MCP-1, and IL-6. This cascade of events leads to the activation of stellate cells, which form a scarring tissue at the damage site initiating fibrogenesis [73]. The mice put on this diet developed steatohepatitis 8 weeks later and after 16 weeks fibrosis had been already formed, affecting the portal and bridging regions of the liver lobules [71].

The diet with methionine and choline deficit is simple to realize and is able to induce a severe form of steatohepatitis in contrast to other diet models. However, this model has serious limitations. Despite its wide usage, it fails to reconstitute the main pathological processes of NAFLD specific to humans including obesity and expressed peripheral resistance to insulin [74]. Besides, mice on the diet lose gradually the body mass (up to 40%) during 8 weeks [75]. It should be also taken into consideration that sensitivity of various mouse lines to this diet differs greatly [76].

Another diet includes the deficit of choline and addition of ethionine. Ethionine is an S-analog of methionine having the ethyl group in place of methyl group in the initial amino acid. It promotes the development of steatohepatitis over a relatively short period of time [77]. Ethionine prevents methylation of macromolecules and has carcinogenic properties. Competing with methionine at the stage of translation, ethionine decreases specific protein activity. High toxicity of ethionine is explained by the need for methionine to implement some biosynthetic and regulatory processes. At the organ level, its supply causes fatty liver (“nutmeg liver”), acute pancreatitis, promotes the development of hepatocellular carcinoma. At the molecular level, ethionine inhibits methylation of tRNA [78]. Unfortunately, this diet model is connected with the body weight loss by the animals and a high mortality rate reaching 60% after 4 months of feeding [79]. In order to reduce lethality and preserve steatosis induction, this diet is alternated with the standard diet in some researches (7% of carbohydrates, 3% of lipids, 50% of polysaccharides, 15% of proteins) [80].

One more diet variant consists in the deficit of only choline with the replacement of proteins with the equimolar mixture of L-amino acids [81]. With such a diet, oxidation of fatty acids is inhibited and lipid synthesis increases leading to the marked oxidative stress, inflammation and liver fibrogenesis. However, it should be noted that histological changes typical for the pathology manifest themselves in the later periods than in case of the diet with the deficit of choline and methionine: steatosis — after 3 weeks, moderate fibrosis — after 21 weeks [82]. Mice on this diet were losing significantly less body weight than being on the previously described diets, but still were not resistant to insulin [83]. This may be regulated by the ration composition (for example, percentage of fat) and duration of feeding. Pedersen et al. [84] have employed the model with choline deficit and showed the steatohepatitis development already after 8 weeks in minipigs. Since minipigs live longer than rodents, the development of obesity and metabolic syndrome is comparable with those going on in humans, which makes it possible to carry out long-term investigations.

The most frequently used model for steatosis induction is the transition of animals to a high-fat diet (45–75% of total calories), which removes the limits of the diet with shortage of methionine and choline since the animals gain the mass and develop peripheral resistance to insulin. The high-fat diet in experimental animals imitates the condition of overeating and hypodynamia, which cause a metabolic syndrome in humans [85]. The application of this diet results in the increased outflow of the non-esterified fatty acids from the fat tissue and proinflammatory cytokines and adipokines are released, which may lead to the ectopic fat deposition in the liver, muscles, and heart [86]. Besides, the high-fat diet initiates the oxidative stress and lipid peroxidation and enhances the work of enzymes of fatty acid β-oxidation.

Apart from steatosis, this model also imitates precisely enough the symptomatology of non-alcoholic steatohepatitis in humans, but it should be noted that in mice it takes about 50 weeks to develop steatohepatitis with weakly expressed fibrosis [87]. Besides, the character of the induced pathology course varies in mice of different lines [88]. For example, C57BL/6 mice are more sensible to the high-fat diet than BALB/c mice. Low sensitivity to the diet in BALB/c is determined by a weak absorption of lipid by the liver and resistance to inflammation development [89]. Mice also have gender differences in the character of obesity-associated metabolic syndrome development [90]. Thus, despite a wide application, a high-fat diet is not the best option for studying NAFLD due to the differences related to the ration composition (the source and nature of fatty acids), mouse lines, gender, and duration of feeding.

An alternative diet with a high content of cholesterol was proposed for rats. This diet induces non-alcoholic steatohepatitis with the signs of fibrosis within 9 weeks [91].

An essential drawback of all enumerated models is only partial imitation of pathological signs typical for a human, which limits interspecies extrapolation of the results [64]. The main disadvantage of the described diets is their failure to develop obesity and resistance to insulin, therefore, in recent years, other dietary patterns were designed permitting the reconstruction of all main aspects of NAFLD pathogenesis.

Recently, a western diet, or a fast-food-diet, was proposed, which represents a modern diet widely used in the industrial countries and is characterized by a high consumption of fats and carbohydrates [92]. Similarly to the human ration, to induce NAFLD in mice, a diet model was designed based on the combination of fats and fructose supplemented in some cases by cholesterol [93]. After six months of the western diet, C57BL/6 mice develop obesity, resistance to insulin, and expressed steatohepatitis [94]. They are also observed to have increased gene expression associated with the development of fibrosis, inflammation, endoplasmic reticulum dysfunction, and triggering lipoapoptosis. All indicated pathological changes are also noted in non-alcoholic steatohepatitis in humans. Besides, Tsuchiya et al. [95] have shown that within 16 weeks, the western diet leads to liver overloading with iron, which often occurs in people with NAFLD. The western diet is a promising direction for modeling fatty liver disease, but presently it is necessary to design a standardized protocol to improve reproducibility of the results.

## *In vitro* models

*In vitro* models are best suitable for express-testing of various drugs or toxic agents. Cellular lines (HepG2, for example), primary cells, spheroids, organoids are referred to the models of this type, and culturing may be performed in culture flasks or using microfluidic technologies. The advantages of the cellular lines consist in their higher replicative capacity compared to the primary cell cultures, isolated directly from tissue, and the possibility of using them for a long period of time. The main drawback of cellular lines for pathology modeling is their immortalized phenotype with an altered metabolic status of the cells [96]. Primary hepatocyte cultures in their turn preserve native morphological and functional characteristics of the cells [97] but show phenotypic instability and low viability [98]. Usage of microfluidic technologies eliminates the disadvantages of the *in vitro* models: the technology allows to maintain the conditions similar to those *in vivo* and control the supply of nutritional and examined substances (drugs, toxins) in order to study the cell response [13].

### Alcohol liver disease

To simulate ALD, several *in vitro* models have been developed [99]. In 2D cultures, hepatocytes quickly lose their phenotype, which limits their application. Now, the most interesting are 3D models, which maintain intercellular contacts, native phenotype, and cell functions for a long time [100]. Deng et al. [101] have used microfluidic technology to study pathophysiological processes in non-parenchymal cells exposed to ethanol. The authors applied endothelial cells EAhy926, stellate cells LX-2, and HepG2. HepG2 were exposed to ethanol (0–150 mM), which significantly enhanced the ROS production in hepatocytes at large concentrations of the toxin (>100 mM) and long-term (>24 h) stimulation. Besides, expression of VE-cadherin decreased indicating the damage of tight contacts in endotheliocytes. While expression of growth factor of vascular endothelia and α-smooth muscle actin (α-SMA), the markers of an early stage of the disease, increased in the LX-2 cells, pointing to the activation of the stellate cells. Corrado et al. [102] have developed a microfluidic biochip with three parallel channels imitating the form of liver sinusoid. The authors investigated the effect of ethanol on the HepG2 cell line. Under the normal conditions, the number of viable HepG2 cells was equal to 65% and albumin production reached 60 ng. Under the action of 100 mM ethanol, there was noted the reduction of culture viability to 55%, albumin production decreased by 3 times. Exposure to 300 mM ethanol reduced the cell viability to as much as 30% and the albumin level to 1 ng. Jang et al. [103] modeled ATP by continuous perfusion of the primary culture of human cells using various ethanol doses (17.4–34.0 mM) within the limits of clinically significant blood alcohol concentrations. The researchers have shown significant elevation of cholesterol level after 48 h of ethanol action. Moreover, there was glycogen accumulation although no glucose release occurred.

### Liver fibrosis

#### Induction by tetrachloromethane

Toxic damage by tetrachloromethane *in vitro* is most frequently modeled using the primary culture of hepatocytes, since significant impairment of the metabolic function takes place in isolated hepatocytes. It has been also shown that protein exchange, protein synthesis, and formation of cAMP are impaired in the isolated hepatocytes, and responses to hormonal stimuli are weak or absent. Concentrations of CCl4 from 0.1 to 0.2 mM in the culture medium cause cytological changes typical for the *in vivo* intoxication model [104]. Generation of 4-hydroxynonenal (toxic aldehyde, the product of lipid peroxidation) takes place, which inhibits the action of adenylate cyclase, cytochrome P450, and ATPase in the cells, but no dystrophic changes of the cells are observed [32]. Inflammation with the release of TNF-α and IL-1β also develops, which leads to the activation of cell apoptosis [105]. Perrissoud et al. [106] have found fast morphological alterations in the primary hepatocyte culture after 5-min exposure to CCl4: degranulation, desquamation of the rough endoplasmic reticulum, and blebbing of the plasmic membrane. Cai et al. [107] exposed the primary rat hepatocyte culture to CCl4 (0.1– 9.0 mM) for 20 h and evaluated the cell viability, which changes depending on the dose. The release of lactate hydrogenase was also observed: its level grew with the increase of the toxin dose. The exposure to CCl4 promoted glutathione depletion, which enhanced with higher doses and cultivation time. The level of tripeptide reduced significantly after the incubation of the cells with 1-mM CCl4 for 8, 12, 16, and 24 h, whereas the levels of MDA and cytochrome C, on the contrary, increased. These results confirm the key role of the hepatocyte oxidative stress in the induction of CCl4 hepatotoxicity leading to their apoptosis.

#### Induction by paracetamol

Exposure to paracetamol contributes to ROS production and reduces the production of reactive nitrogen species in the *in vitro* models. Using the U937 culture (a promonocytic cell line isolated from the histiocytic lymph), Jamil et al. [108] have established that paracetamol at the 0.01– 100.0 mM concentration increases the content of H2O2 and other ROS leading to the oxidative stress, whereas NO generation decreases. Another study [109] has shown that after the action of the preparation on the mouse hepatoma cells Hepa 1-6 at the concentrations of 50, 100, and 300 mM, ROS production by mitochondria enhances. The increased amount of ROS contributes to activation of lipid peroxide: after the exposure of the rat hepatocytes to paracetamol at 100-mM concentration for 1 h, the amount of malondialdehyde, an intermediate product of lipid peroxidation, increased. There were also changes in the antioxidant content [110]. In their study, Bader et al. [111] described the action of paracetamol at the concentration of 6 and 15 mM for 24 h on the culture of the embryonic rat liver cells. The toxin decreased significantly the total glutathione level but enhanced telomerase activity. It is reported in another study that the glutathione level was also significantly reduced on the primary culture of the rat hepatocytes after the action of various paracetamol concentrations (7 nM, 12, and 100 mM) for 24, 22, and 1 h, respectively [112].

Pierce et al. [113] incubated a mouse hepatocyte culture with different concentrations of paracetamol (1– 10 mM) and revealed initiation of apoptosis and necrosis mediated by generation of NAPQI-reactive metabolite after 48–72 h of cultivation.

Clarke et al. [114] evaluated paracetamol hepatotoxicity using microfluidic technologies. Significant reduction of albumin concentration has been shown to occur on day 6 of cultivation under the action of 10 mM of preparation as compared to the control group (0 mM) with 1-mM concentration. Foster et al. [115] also employed microfluidic technology with a primary human hepatocyte culture, which was incubated with paracetamol (0–10 mM); a dose-dependent cytotoxicity of the preparation has been demonstrated. The content of albumin in the liver cells already decreases greatly after 24 h of exposure. The dose-dependent reduction of albumin secretion by hepatocytes and cell viability continued until the 10^th^ day of cultivation. Rodimova et al. [116] explored the change of the metabolic status of the primary mouse hepatocyte culture under the action of paracetamol using microfluidic technology and fluorescent-lifetime imaging microscopy (FLIM). There has been found the reduction of general metabolic activity of hepatocytes and the development of their mitochondrial dysfunction, which led to the diminished ATP content in the cells.

Prot et al. [117] investigated transcriptomic and proteomic-metabolomic profiles in the microfluidic HepG2/C3a model under the action of 1-mM paracetamol. The toxic effect of the preparation impaired lipid exchange and calcium homeostasis in the cells via the pathway of VDR/RXR activation. Furthermore, DNA damage, cell cycle arrest, apoptotic and necrotic cell death, reorganization of cytoskeleton were observed. The researchers have demonstrated the change of mitochondrial membrane potential and the development mitochondrial dysfunction. The authors managed to reconstitute the pathways of glutathione depletion via NAPQI generation.

The specific consumption increase of cysteine, histidine, and methionine in the cells cultivated on microfluidic chips correlates with the intensity of the glutathione pathway, which is involved in detoxification of paracetamol. Under metabolic stress, 2-hydroxibutirate is also released as a by-product of cystathionine splitting to cysteine before its conversion to glutathione [118]. Besides, S-adenosyl methionine, the glutathione precursor, is generated by conjugation of methionine with ATP and cysteine participating in the glutathione synthesis. The elevated level of these molecules in the medium in the process of cultivating primary hepatocytes with paracetamol is a marker of toxic cell damage. However, the researchers did not find any accumulation of taurine, creatine, or ophthalmic acid in the medium during cultivation of primary hepatocytes on microfluidic chips, which are identified in the urine test and liver extracts in the process of *in vivo* investigation [119]. The elevation of 3-hydroxibutirate level in the medium with paracetamol during cultivation of primary hepatocytes on microfluidic chips points to the enhancement of lipid metabolism by the ketolytic pathway. When the glutathione pathway is activated in the cells cultivated in the medium with paracetamol on chips, increased expression of the genes *ggt7*, *g6pd*, *gpx2*, *gpx3*, *gstm2/4*, *gstt2* and production of the G6PD and TXNRD1 proteins occurred relative to the control. Moreover, intensive activation of the tricarbonic acid cycle was observed in the cells due to the increased consumption of glutamine, glucose, and fructose, and lactate production [120].

### Steatosis

2D cultures of hepatocytes, pathological processes in which are induced by the addition of free fatty acids (mainly oleic and palmitic (2:1)) to the culture medium, are widely used for NAFLD modeling [121]. The addition of fatty acids to the medium with hepatocytes leads to accumulation of TG in the cytoplasm causing stress to endoplasmic reticulum and cell death [122]. Fatty dystrophy of the liver cells may also be induced with bisphenol A, which increases lipid accumulation due to the SREBP1 upregulation or valproate augmenting absorption of fatty acids and TG synthesis [123]. Hepatocyte 2D monoculture enables to study the changes of metabolic pathways in these cells [124]. However, the main disadvantage of such models is the lack of interaction with non-parenchymal cells, which are important for initiation of inflammatory processes associated with fibrosis pathogenesis in NAFLD. Co-cultivation of hepatocytes and non-parenchymal cells makes it possible to overcome a number of limitations. One of such models is co-cultivation of hepatocytes with stellate liver cells. Barbero-Becerra et al. [125] have designed a model of co-cultures Huh7 (the cell line of human hepatocytes) and LX-2 (the line of human stellate cells). The effect of fatty acids on the co-culture has been demonstrated to induce the α-SMA expression (a marker of activated fibroblasts) in the LX-2 cells only with concurrent cultivation with Huh7. Activation of stellate cells did not depend on the accumulation of fatty acids but required interaction with hepatocytes.

Cultivation of primary hepatocytes together with Kupffer cells or endothelial cells serves as an effective tool for evaluation of mechanisms by which fatty acids trigger inflammatory processes in the liver [126]. Co-cultivation of primary hepatocytes, stellate liver cells, and human Kupffer cells and addition of fatty acids, glucose, insulin, and inflammatory cytokines into the medium for imitation of non-alcoholic fatty hepatitis has shown increased lipogenesis *de novo* [127]. An oxidative stress, inflammation, activation of stellate cells, and triggering of the fibrotic process were also observed in this model [128]. Chen and Ma [129] cultivated HepG2 cells with THP-1 macrophages. Macrophages were placed on the slides, which were then put in the 6-well plates with HepG2 culture and fatty acids were added at the 1 mmol/l concentration (palmitic and oleic acids (1:2)). The pathogenetic signs of non-alcoholic fatty liver disease were established 24 h after the beginning of induction. The main drawback of such co-cultivation is a complicated selection of optimal conditions and culture medium for maintaining various cell populations [130].

Microfluidic technology was used for creation of functional liver-on-a-chip constructs. The liver microarchitecture was imitated using a polymer scaffold consisting of hundreds of tiny channels with preservation of dynamic tissue properties [131]. The cells were supplied with nutrients and oxygen with subsequent elimination of metabolites, which imitated microcirculation in the liver [132]. Cultivation of HepG2 cells with a mixture of free fatty acids (oleic and palmitic acids (2:1)) on a microfluidic chip provided gradual accumulation of lipids, with the chip effectively maintaining hepatocyte metabolic activity and viability and reducing oxidative stress as compared to static cultures [133]. Kostrzewski et al. [134] incubated the primary hepatocyte culture with free fatty acids (600 μM) and observed gradual 4-fold increase of the TG level and elevated activity of cytochromes P450: 2E1 and 7A1 (involved in cholesterol metabolism), IGFβ1 (a marker of metabolic disorders in the liver), PDK4 (provides liver resistance to insulin), and FABP1 (a fatty acid-binding protein). CYP3A4 activity was reduced by more than 40%, and CYP2C9 — by 30%, which agrees with the results of the investigations using human hepatocytes and clinical specimens taken from patients with NAFLD. Another study [135] reported the influence of lipid accumulation on the secretion of adipokines, proteins connected with fibrosis and damage development (fibrinogen, TIMP-1), and inflammatory markers (IL-8, MIF).

Lee and Sung [136] have designed an intestine– liver-on-a-chip model for studying the interaction of these organs in the context of NAFLD, where fatty acids were absorbed through the intestine layer with subsequent secretion of chylomicrons, which contributed to TG accumulation in hepatocytes. Freag et al. [137] perfused hepatocytes, Kupffer cells, and stellate cells with a high concentration of free fatty acids via a single-layer microfluidic channel for 10 days. The exposure to a high concentration of fatty acids resulted in significant accumulation of neutral lipids inside the stellate liver cells in comparison with the cells cultivated in physiological conditions. Moreover, the lactate dehydrogenase activity grew relative to the control already on the second day.

Under *in vivo* conditions in the process of nonalcoholic steatohepatitis progression, the quiescent stellate cells are quickly activated into myofibroblasts, moreover, the expression of α-SMA, which is one of the indicative markers of stellate liver cell activation, sharply increases [138]. When modeling this process using the system-on-chip, hyperexpression of *α-sma* has been noted in the stellate cells with 8-fold increase of the activated cells [139].

The microfluidic technology essentially widens the possibilities of NAFLD modeling in the *in vitro* conditions including modeling of the interaction between the liver and other organs, which makes it an effective tool for studying pathology-associated metabolic changes [97].

## *Ex vivo* models

The *in vivo* and *in vitro* models, which are given preference in the majority of investigations, have been considered above. However, it is important to note that *in vitro* models do not provide full imitation of complicated multifactorial pathogenesis and progression of the disease [140]. Complex pathological mechanisms in the liver are difficult to model using one type of cells since this organ represents a sophisticated system consisting of various tissue types including parenchymal and stromal components. Besides, employment of animal models is unable to fully reflect the work of the human immune system, which is a significant drawback, since the development of inflammation is one of the key aspects of liver disease pathogenesis [141]. A number of these limitations may be overcome by using *ex vivo* models of liver slices, the universal models, which may preserve the tissue architecture and its microenvironment reconstituting thereby the conditions close to native. Moreover, constant gas diffusion and access to the medium nutrients are also maintained. Liver slices are an easily reproducible model providing the viability of hepatocytes and other liver cells for 3–5 days; the preservation of liver slides for 15 days has been reported. The application of this model makes it possible to analyze simultaneously the effect of different medicinal and toxic agents, and also to model pathological processes collecting specimens from one animal, which minimizes the contribution of individual differences in animals. In addition, co-cultivation of liver slices with autologous cells of the peripheral blood enables researches to reconstitute complex interactions of the immune system and liver tissue [142].

### Alcoholic liver disease

Application of the *ex vivo* models helped greatly understand ALD pathogenesis in a human. Klassen et al. [143] have found that ethanol exposure causes tissue damage, development of steatosis, and oxidative stress. Moreover, complete ethanol metabolization occurs some days after the initial action. The authors incubated liver slices taken from rats in the medium with 25 mM ethanol and in the medium with the inhibitor of ethanol metabolism, 4-methylpyrasol (0.5 mM). The addition of ethanol to the culture medium with liver slices has been found to induce pathological processes similar to those running in *in vivo* models of chronic ethanol consumption. Thus, ethanol oxidation in the hepatocytes with acetaldehyde generation, increased lipogenesis, and changes in the intracellular redox status, as well as the reduction of albumin secretion have been shown to occur in the liver slices [144].

Palma et al. [29] studied the effect of ethanol on the morphology, structure, and function of mitochondria and have shown that megamitochondria, which are also typical for ALD pathogenesis, are generated under the action of ethanol in the dose of 50–250 mM for 24–72 h. The appearance of megamitochondria during ALD development was detected as early as in 1970s [145]. In the recent studies, Altamirano et al. [5] have established that the appearance of megamitochondria in the liver cells determines a favorable outcome in patients with severe alcoholic hepatitis. At the same time, Teli et al. [146] in the earlier investigation interpreted the appearance of megamitochondria in patients with alcoholism as a bad prognostic sign and connected their presence with a higher risk of liver fibrosis/cirrhosis progression. Thus, presently, the role of megamitochondria in the development of pathological changes caused by ethanol is not fully studied. Megamitochondria are supposed to participate in defending hepatocytes against toxic damage and to be one of the components of the compensatory processes during ADL development. Palma et al. [147] using liver slices investigated molecular cascades associated with the development of megamitochondria in hepatocytes and defined a key role of mitochondria-shaping proteins (MSP). The authors cultivated liver slices harvested from the liver of a healthy human for 24 h with different ethanol doses (50, 100, and 250 mM). The toxic effects were assessed by quantitative determination of the ATP level in the cells. In the control liver slices, the ATP concentration was within the range of 3–12 nmol per 1 mg protein and remained stable for 5 days. The action of ethanol in three indicated doses resulted in the reduction of the ATP level relative to the control [147]. The electron microscopy has detected the signs of cell damage with the presence of cytoplasm cariorexis, expressed vacuolization, and the loss of the membrane structural integrity. A direct correlation was also found between the size enlargement of the mitochondrial liver cells and the ethanol doses. In another study [148], the tendency to hyperactivation of the Drp1-cascade associated with mitochondria division in the liver of the patients with alcohol hepatitis has been noted. Significant hyperactivation of this cascade was accompanied by apoptotic death of hepatocytes and a severe course of the disease [149].

### Liver fibrosis

#### Induction by paracetamol

Granitzny et al. [150] used rat liver slices to study the toxic effect of paracetamol. The researchers added various paracetamol concentrations (2.55–15 mM) into the culture medium and incubated the liver slices for 24 h assessing thereafter the ATP content. A gradual increase of the ATP synthesis reached its maximum in the liver transplant at the 5.5-mM paracetamol concentration. Higher concentrations (10–15 mM) reduced sharply the viability of the cells in the tissue transplants. The researchers evaluated a number of other markers. Glutamate dehydrogenase showed a dose-dependent reduction in the whole range of concentrations, whereas the activity of aspartate aminotransferase varied depending on the added preparation concentrations. Production of urea and albumin increased gradually and reached its maximum at 5.5 mM, which correlated with the growth of ATP content. At 10–15 mM paracetamol concentrations, necrosis was noted to develop in the liver slices, though small tissue areas remained viable.

The molecular and genetic analysis using the models of liver slices conducted by Ji et al. [151] have revealed the increased expression in 13 genes associated with a TGF-β1 cascade when exposed to paracetamol. The TGF-β1 cascade plays a key role in the development of liver fibrosis. Li et al. [152] incubated rat liver slices with paracetamol at 500-μM concentration for 6 h. The concentration of glutathione and lactate dehydrogenase was found to decrease significantly relative to the control group. Liver slices are usually obtained from rats; however, it should be taken into consideration that rats are less susceptible to paracetamol action as compared to mice even if the doses are high [153].

In their study, Dewyse et al. [154] have shown that paracetamol activates stellate cells in the liver slices similar to the *in vivo* model but full development of fibrosis is not observed.

A perfusion model is one more *ex vivo* model, which was applied by Schreiter et al. [155]: human liver specimens were perfused with a toxin for 30 h in a closed-loop mode, in a specified time intervals perfusate samples were taken for the blood gas analysis, search for the markers of hepatocyte injury, and evaluation of the liver synthetic capacity. Perfusion *ex vivo* imitates the supply of fluid to the organ or tissues via the lymphatic or blood circulatory system. In this model, metabolism specific to the organ comparable with the *in vivo* model is maintained by supplying nutrients and oxygen at the necessary concentration. The authors also determined clearance of indocyanine green (ICG) from the perfusate for functional liver evaluation after 4, 20, and 28 h of perfusion. The clearance parameters have been shown to be sufficiently stable, indicating the preservation of the liver function and metabolic activity of hepatocytes during the entire period of perfusion. After the perfusion of the liver specimens with paracetamol for 30 h, the period of ICG elimination increased by 3 times. Besides, the preparation action reduced significantly the amount of glucose consumed by the cells and production of lactate relative to the control. The rate of albumin and urea synthesis was nearly equal during the entire period of perfusion both with and without paracetamol. Marked damage to hepatocytes have been shown to occur under the action of paracetamol, which is seen by the increase of glutamate dehydrogenase, cytokeratine-18, aspartate and alanine aminotransferase, mitochondrial DNA. Thus, the perfusion liver system is an effective model reflecting the diversity of the individual human response to the action of the preparation, and gives a more complete notion about the mechanisms of paracetamol pathogenesis. However, inability to maintain the viability of liver specimens for a long time prevents using the model for a long-term testing of the preparation effect [156].

#### Induction by tetrachloromethane

In their study, van de Bovenkamp et al. [157] analyzed expressions of molecular markers of stellate liver cells and found that expression of α/β-crystalline, *klf6*, and *hsp47* elevates when exposed to CCl4. Expression enhancement correlated with dose and duration of toxin action.

Increased expression of 19 genes (*jun*, *litaf*, *mapk6*, *plat*, *serpine1* being among others) involved in the TGF-β1 pathway has been also shown, which points to triggering the fibrotic process (after 16 h) under the action of tetrachloromethane. TGF-β has been found to induce *serpine1* gene expression by pSmad2L/C and promote the deposit of extracellular matrix components by myofibroblasts (activated stellate cells) [158]. Although it should be noted that the toxin does not dissolve in the culture media and therefore toxic damage is modulated by saturation with phase vapors over the medium surface, the toxin vapors in this case diffuse freely into the medium. The exact concentration in the medium is therefore difficult to determine.

### Steatosis

The development of intrahepatic inflammation, as well as a complex interaction of all types of liver cells, are important aspects of NAFLD pathogenesis in a chronic form. Native immune cells remain in the liver slices and the signal pathways of interaction between hepatocytes, Kupffer cells, and stellate liver cells are maintained, which makes it possible to model complex NAFLD pathogenesis [159]. Palma et al. [29] added free fatty acids (a mixture of oleic and linoleic acids; 0.1-mM solution) into the culture medium with human liver slices and noted lipid infiltration of the liver cells with triggering lipotoxicity, which corresponded to the signs observed in patients at the early stages of the pathology.

The source of liver slices influences the character of steatosis course and lipid metabolism. For example, Graulet et al. [160] have shown that TG accumulation in cytosol of the rat and calf hepatocytes differs at equivalent doses of oleic acid. The capacity of hepatocytes to synthetize TG from fatty acids added to the medium with liver slices obtained from the cattle, sheep, pig, guinea pig, rat, and rabbit was studied in another work. The intensity of lipogenesis in all animals has been shown to exhibit significant differences. Thus, fatty dystrophy was induced less effectively in the slices of the cattle, pig, and guinea pig [161].

One more characteristic pathological change in NAFLD is a metabolic syndrome accompanied by hyperlipidemia, hyperinsulinemia, and hyperglycemia [162]. To reconstitute the metabolic syndrome, mouse liver slices were cultivated in the medium with glucose, fructose, insulin, and palmitic acid at supraphysiological concentrations. After 48 h of incubation, the viability (ATP content) of the liver cells was not significantly reduced. Moreover, no influence of palmitic acid on the ATP synthesis was also found, which might be explained by a high activity of antioxidant defense in the specimens or insufficient palmitate concentration to initiate mitochondrial dysfunction [163]. The molecular mechanism of palmitate lipotoxicity has not been sufficiently studied, but it is likely that β-oxidation of fatty acids and generation of free radicals play here an important role [164]. This group of investigators managed to induce a pathogenetic phenotype in the mouse liver slices in the medium containing 36 mM of glucose, 5 mM of fructose, 240 μM of palmitate, 480 μM of oleate, and 100 nM of insulin, which led to the 2-fold increase of TG after 48 h as compared to the control group. Besides, the intracellular ATP content has also reduced the viability of the liver cells. In order to exclude the reduction of viability, Prins et al. [165] minimized the concentration of saccharides and fatty acids. No difference in TG accumulation was observed when the glucose concentration was reduced from 36 to 11 mM; 1 mM of fructose was enough to reach the maximum TG accumulation after 24 and 48 h. The concentration of palmitate and oleate also reduced to 120 and 240 μM, respectively. After 48 h of incubation in the GFIPO medium, the TG level elevated by 67% relative to the control. Significant increase of acylcarnitines C14-C18 confirmed that palmitate andoleate were absorbed by the liver cells. In order to differentiate the effects of fatty acid generation and lipogenesis *de novo*, conditions were changed (5 mM of fructose and 0 mM of fatty acids, GFI medium), which promoted less TG accumulation relative to GFIPO. When cultivated in the GFI and GFIPO media, the levels of transcripts of proteins of fatty acid synthesis and absorption of hexoses Elovl6, Fasn, and Slc2a2 were elevated, while in the GFI medium, expression of acetyl-CoA-carboxylase and acyl-CoA-desaturase was additionally elevated. There was also reduced expression of the genes of fatty acid oxidation enzymes and elevated expression of osteopontin, a marker of liver fibrosis.

The application of the *ex vivo* models has some limitations connected with damage or loss of natural tissue architecture when harvesting slices. Model viability depends on the tissue quality, the protocol used, and equipment to maintain optimal conditions. Moreover, the *ex vivo* models imply the one analysis–one specimen ratio, preventing the repeated use of the specimen to monitor the changes. Finally, these models are not preferable if metabolic changes are to be analyzed directly in hepatocytes since the interpretation of the results is complicated by the influence of the surrounding non-parenchymal cells [166].

## Conclusion

In the present review, we have considered the most common *in vivo*, *in vitro*, and *ex vivo* models for studying non-alcoholic fatty and alcoholic liver disease, toxic liver damage, and fibrosis, described their merits, limitations, and usage perspectives. Great emphasis was made on the mechanisms of pathological condition development in each model, and on the possibility of reconstruction of various key aspects of pathogenesis for all mentioned pathologies. Despite all advantages and drawbacks, presently, there is no consensus on the choice of the most adequate models for studying liver pathology. This choice is determined by specific goals and tasks of the experiment.
